# Supplementary figures and images for: Distinct but overlapping roles of LRRTM1 and LRRTM2 in developing and mature hippocampal circuits
Source: eLife. 2022 Jun 6;11:e64742. doi: 10.7554/eLife.64742 (PMC9170246; doi:10.7554/eLife.64742)

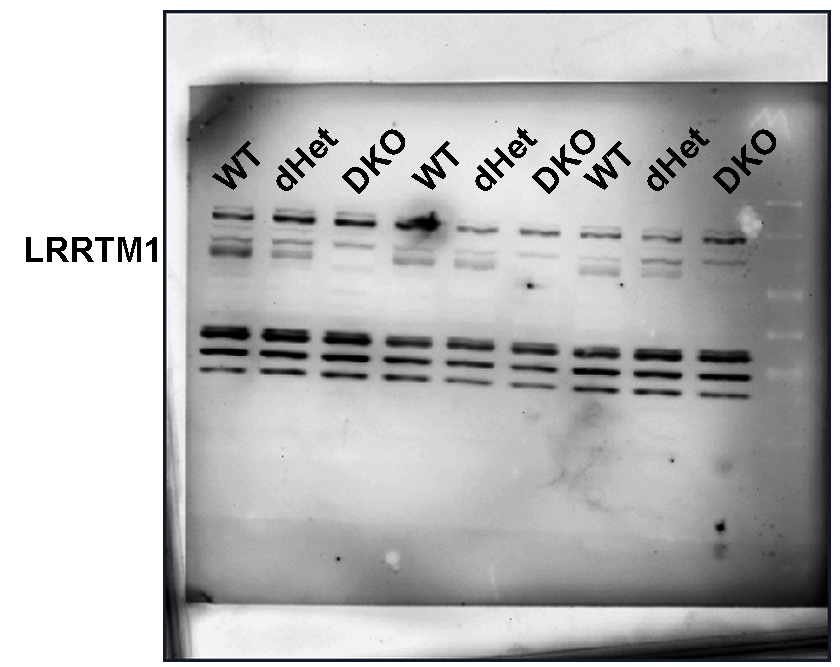

Supplement: Figure 1—figure supplement 1—source data 1. [file elife-64742-fig1-figsupp1-data1.zip › Figure 1 - Figure supplement 1 - source blot 1.tif]

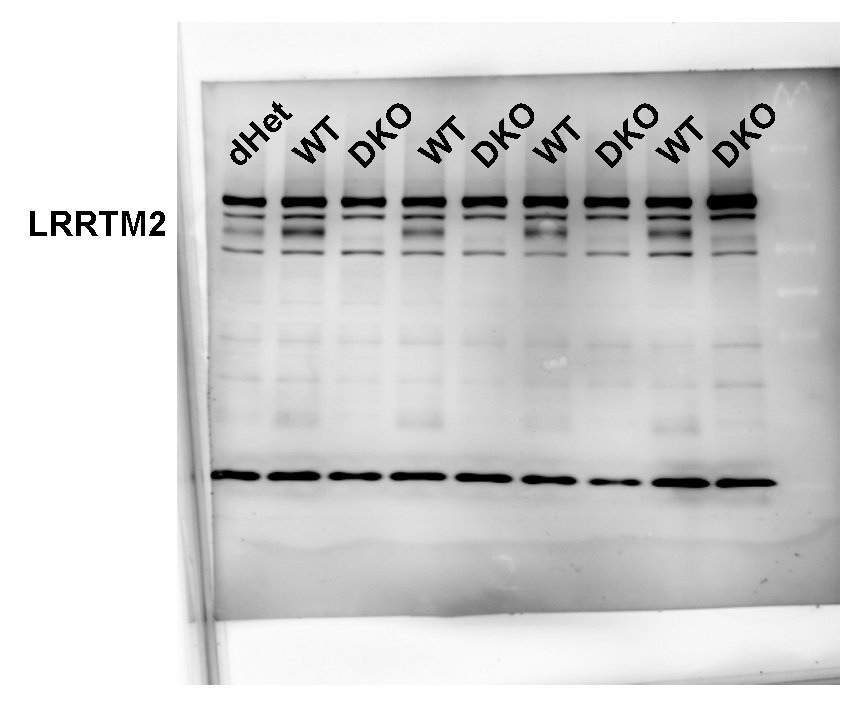

Supplement: Figure 1—figure supplement 1—source data 1. [file elife-64742-fig1-figsupp1-data1.zip › Figure 1 - Figure supplement 1 - source blot 2.tif]

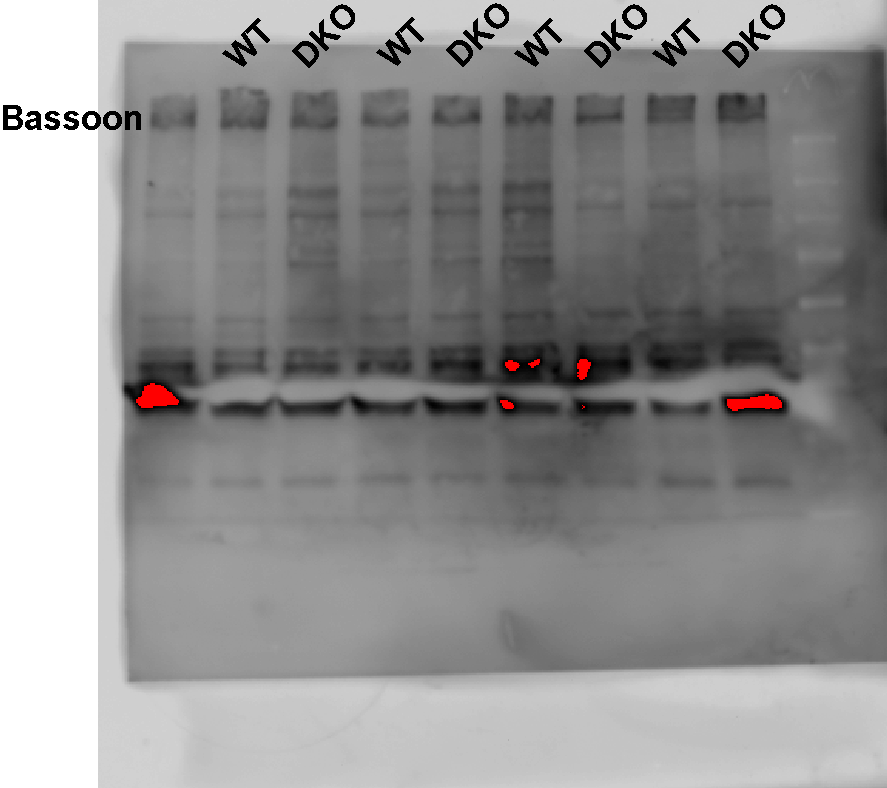

Supplement: Figure 1—figure supplement 2—source data 1. [file elife-64742-fig1-figsupp2-data1.zip › Bassoon original blot.tif]

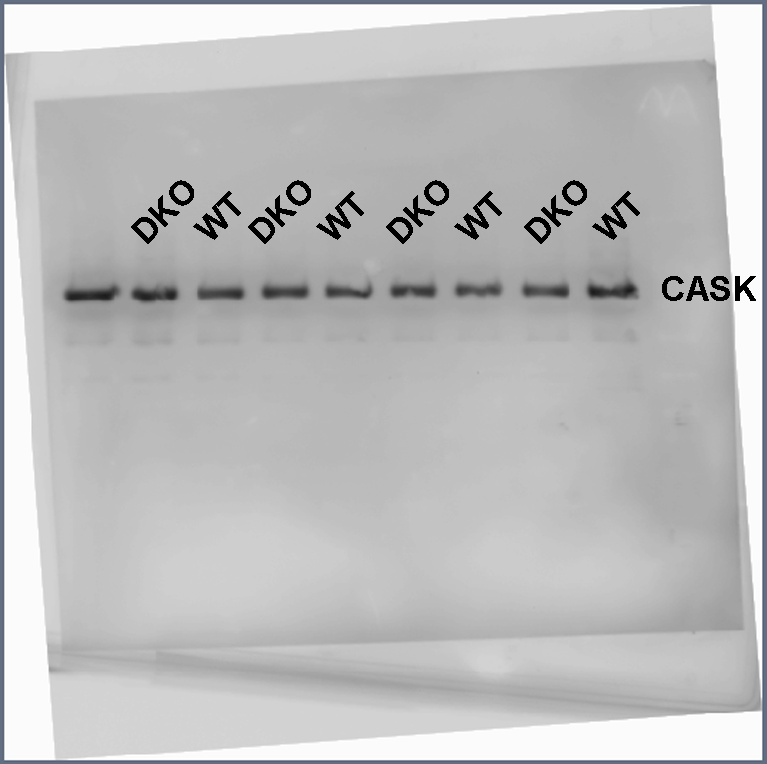

Supplement: Figure 1—figure supplement 2—source data 1. [file elife-64742-fig1-figsupp2-data1.zip › CASK original blot.tif]

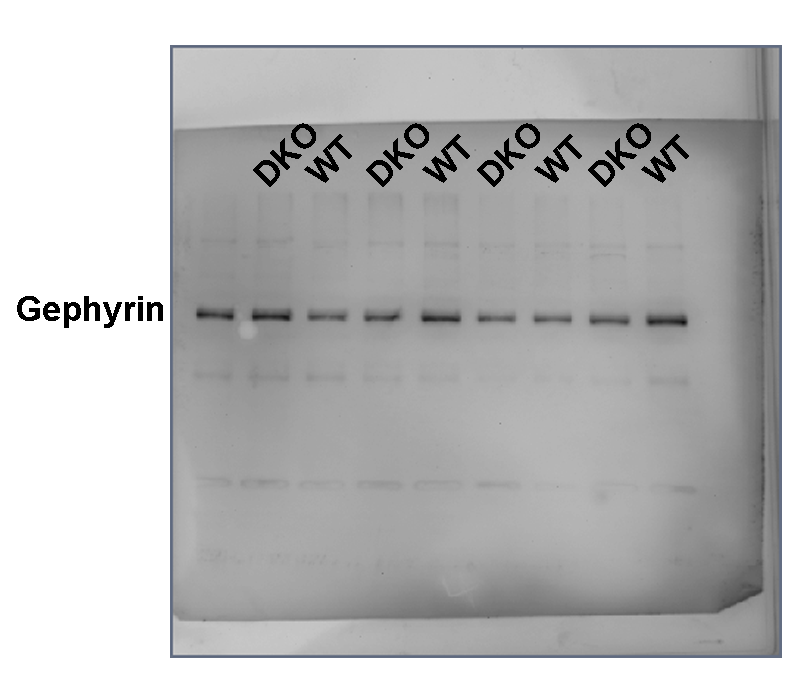

Supplement: Figure 1—figure supplement 2—source data 1. [file elife-64742-fig1-figsupp2-data1.zip › Gephyrin original blot.tif]

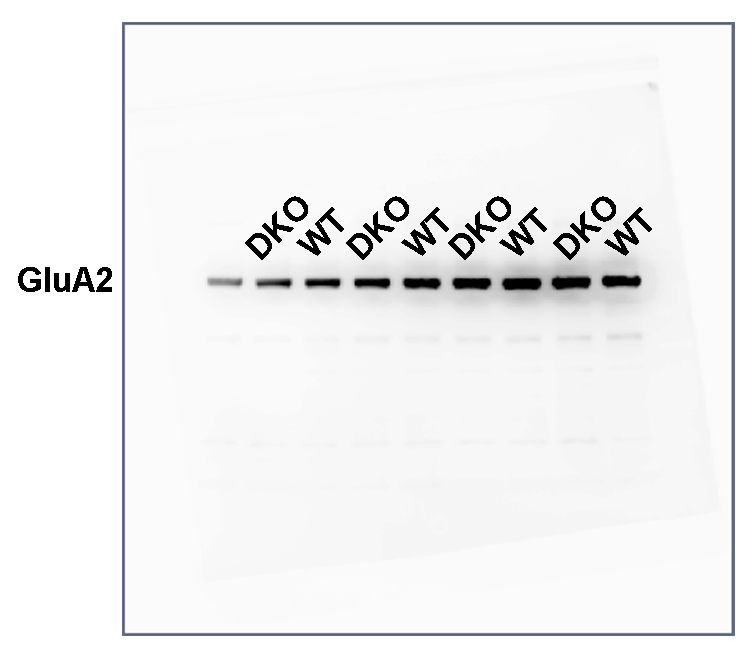

Supplement: Figure 1—figure supplement 2—source data 1. [file elife-64742-fig1-figsupp2-data1.zip › GluA2 original blot.tif]

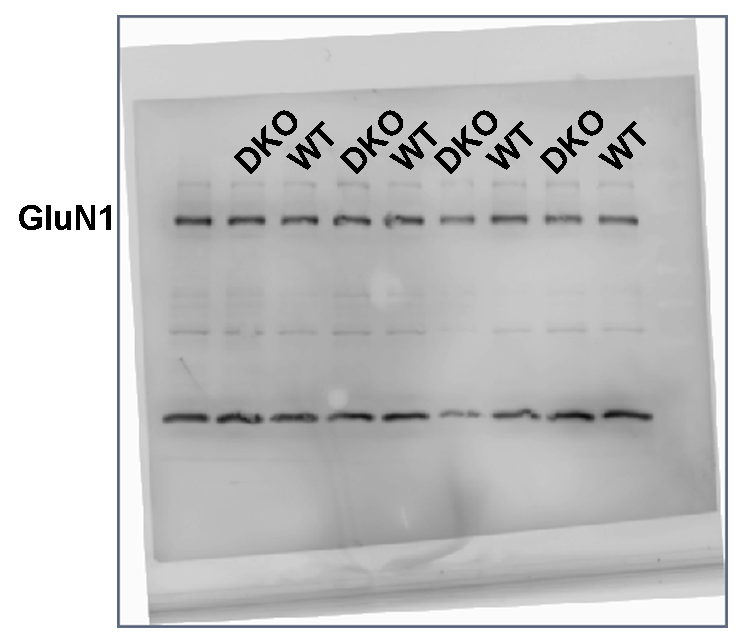

Supplement: Figure 1—figure supplement 2—source data 1. [file elife-64742-fig1-figsupp2-data1.zip › GluN1 original blot.tif]

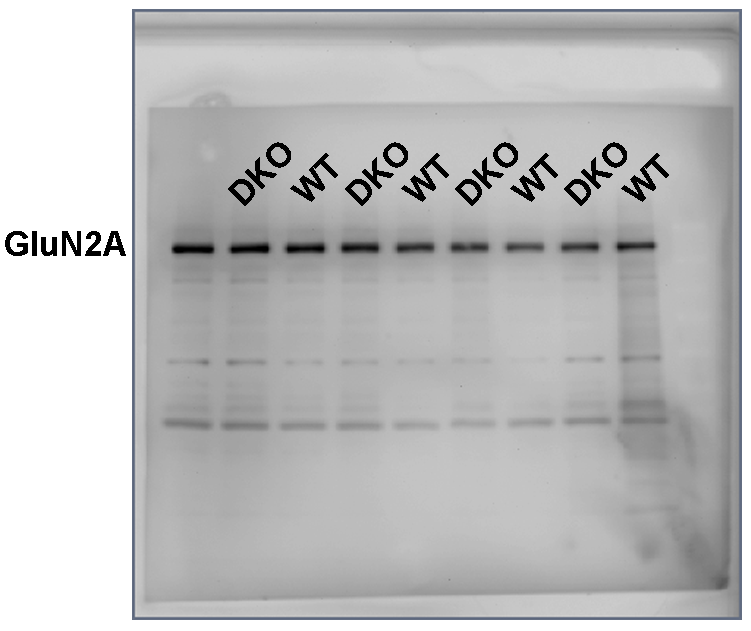

Supplement: Figure 1—figure supplement 2—source data 1. [file elife-64742-fig1-figsupp2-data1.zip › GluN2A original blot.tif]

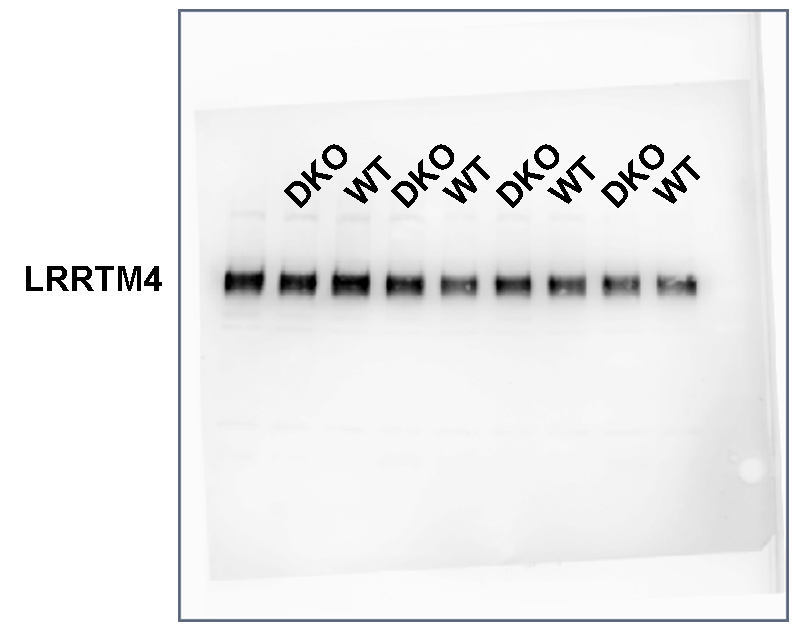

Supplement: Figure 1—figure supplement 2—source data 1. [file elife-64742-fig1-figsupp2-data1.zip › LRRTM4 original blot.tif]

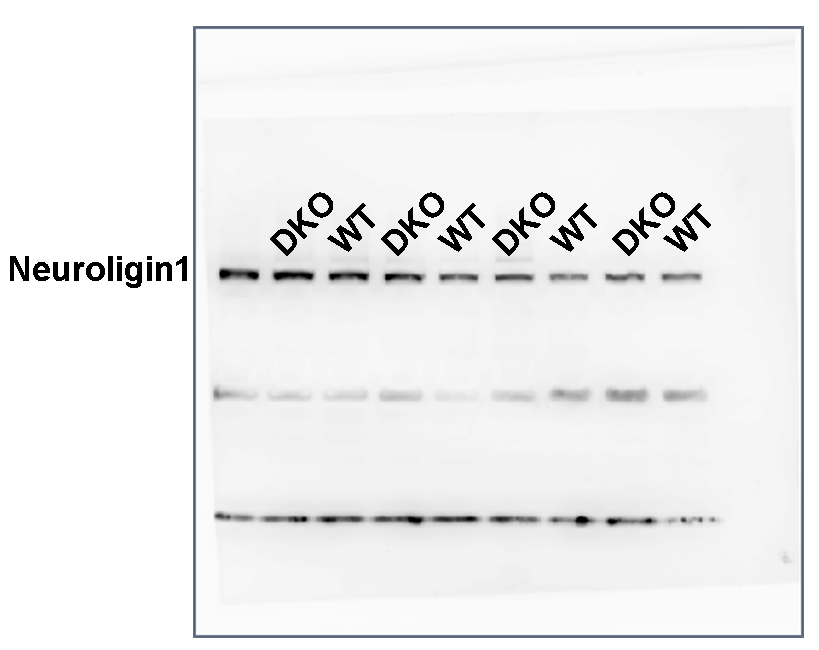

Supplement: Figure 1—figure supplement 2—source data 1. [file elife-64742-fig1-figsupp2-data1.zip › Neuroligin1 original blot.tif]

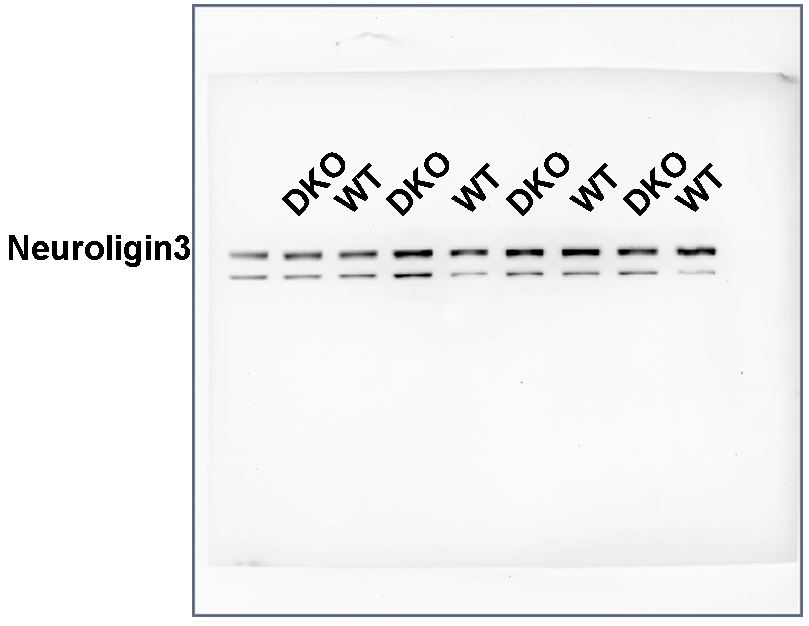

Supplement: Figure 1—figure supplement 2—source data 1. [file elife-64742-fig1-figsupp2-data1.zip › Neuroligin3 original blot.tif]

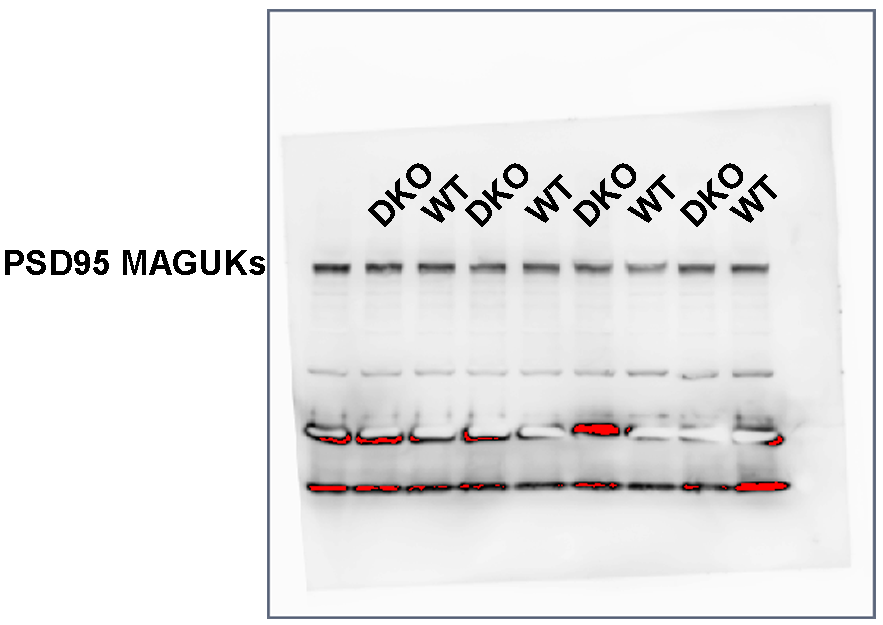

Supplement: Figure 1—figure supplement 2—source data 1. [file elife-64742-fig1-figsupp2-data1.zip › PSD95-MAGUKs original blot.tif]

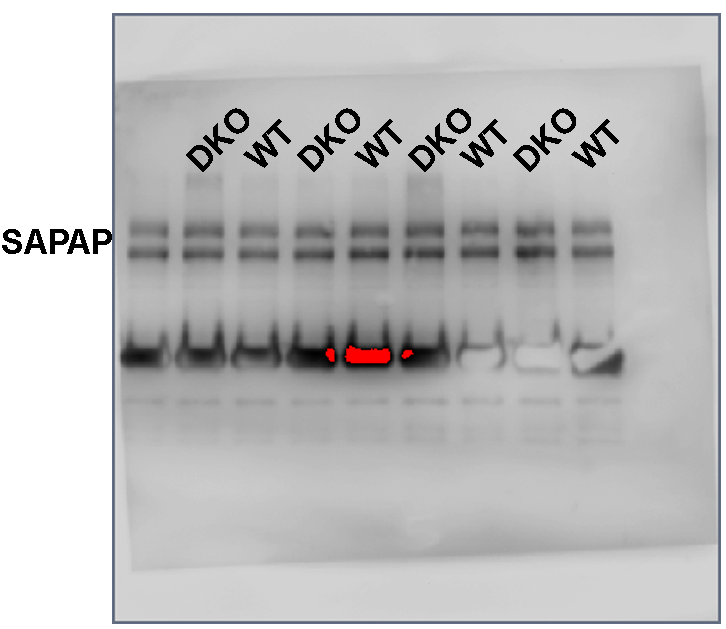

Supplement: Figure 1—figure supplement 2—source data 1. [file elife-64742-fig1-figsupp2-data1.zip › SAPAP original blot.tif]

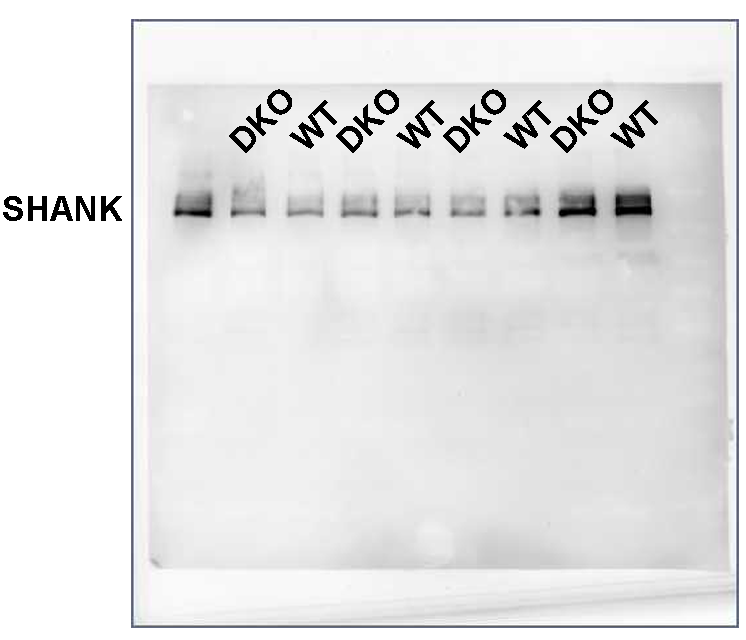

Supplement: Figure 1—figure supplement 2—source data 1. [file elife-64742-fig1-figsupp2-data1.zip › SHANK original blot.tif]

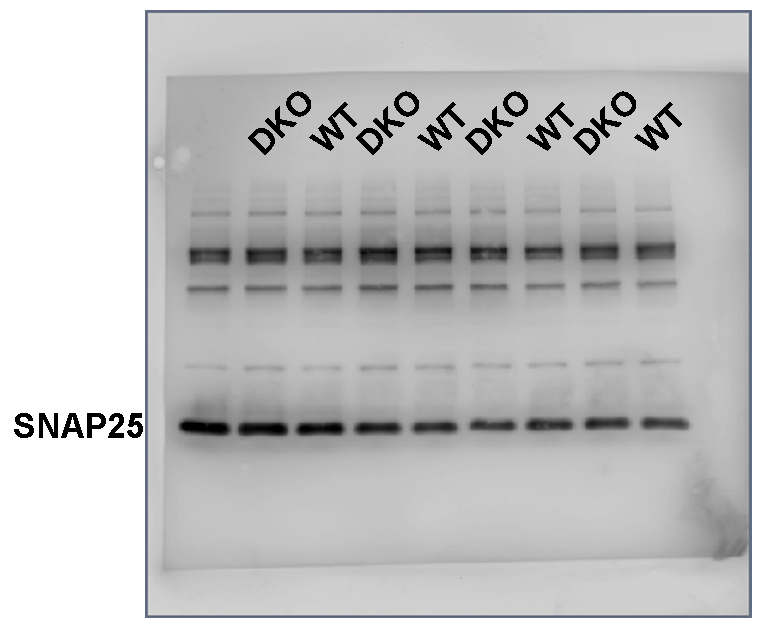

Supplement: Figure 1—figure supplement 2—source data 1. [file elife-64742-fig1-figsupp2-data1.zip › SNAP25 original blot.tif]

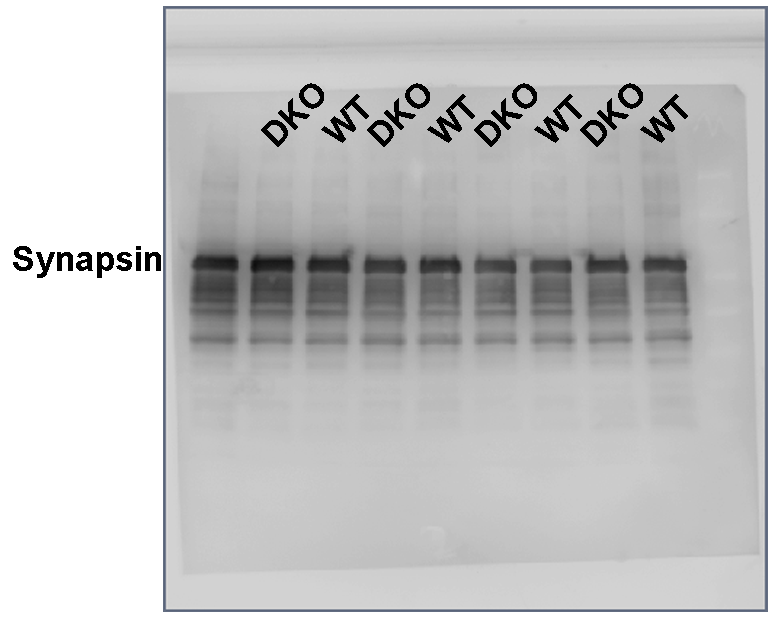

Supplement: Figure 1—figure supplement 2—source data 1. [file elife-64742-fig1-figsupp2-data1.zip › Synapsin original blot.tif]

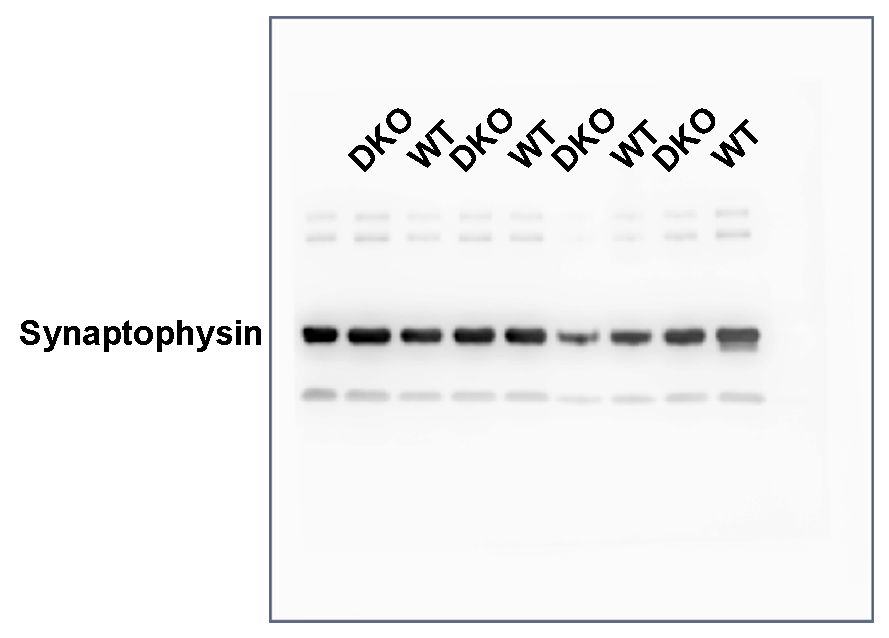

Supplement: Figure 1—figure supplement 2—source data 1. [file elife-64742-fig1-figsupp2-data1.zip › Synaptophysin original blot.tif]

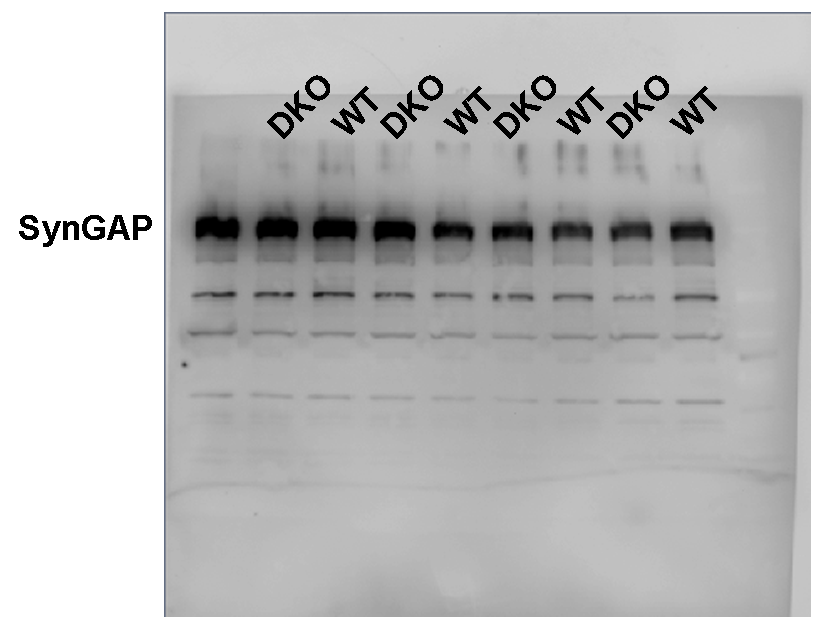

Supplement: Figure 1—figure supplement 2—source data 1. [file elife-64742-fig1-figsupp2-data1.zip › SynGAP original blot.tif]

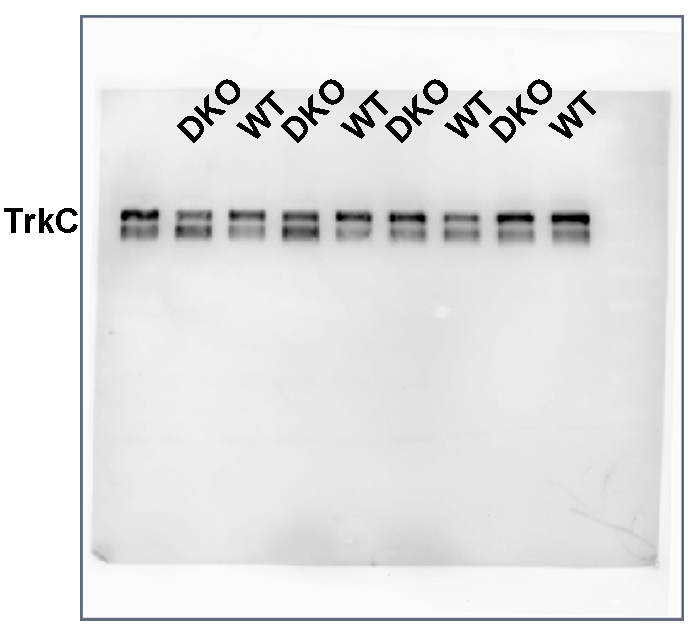

Supplement: Figure 1—figure supplement 2—source data 1. [file elife-64742-fig1-figsupp2-data1.zip › TrkC original blot.tif]

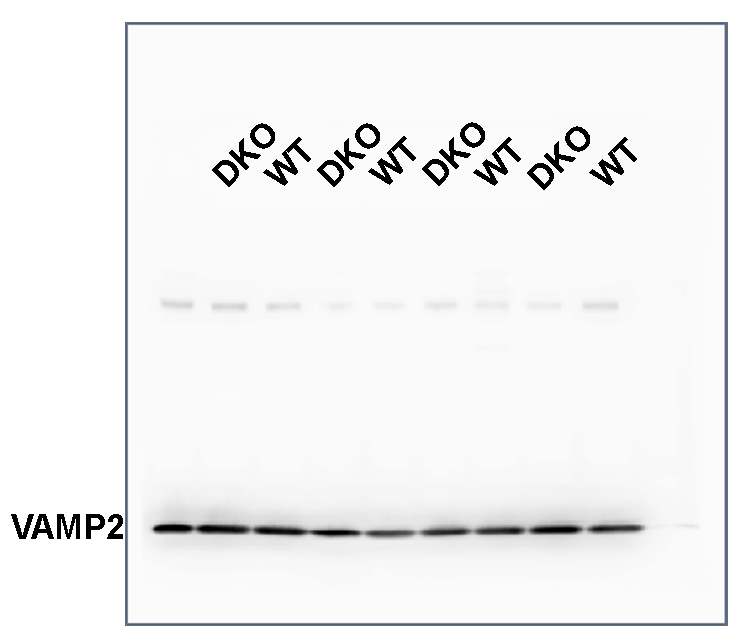

Supplement: Figure 1—figure supplement 2—source data 1. [file elife-64742-fig1-figsupp2-data1.zip › VAMP2 original blot.tif]

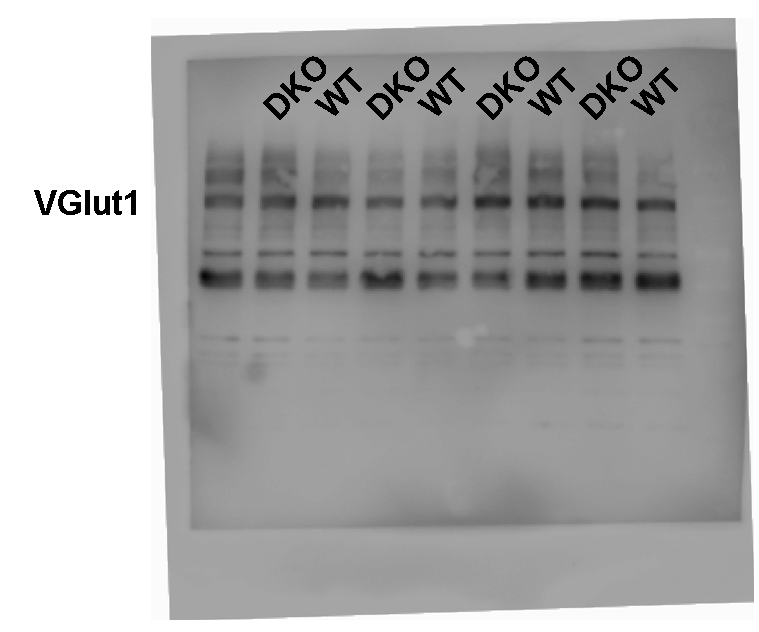

Supplement: Figure 1—figure supplement 2—source data 1. [file elife-64742-fig1-figsupp2-data1.zip › VGlut1 original blot.tif]
