# Supplementary material for: Distinct but overlapping roles of LRRTM1 and LRRTM2 in developing and mature hippocampal circuits
Source: eLife. 2022 Jun 6;11:e64742. doi: 10.7554/eLife.64742 (PMC9170246; doi:10.7554/eLife.64742)
Supplement: Transparent reporting form [file elife-64742-transrepform1.pdf]

eLIFE

1st Floor
24 Hills Road
Cambridge CB2 1JP, UK

P 01223 855340
W elifesciences.org
T @elife

eLife's transparent reporting form

We encourage authors to provide detailed information *within their submission* to facilitate the interpretation and replication of experiments. Authors can upload supporting documentation to indicate the use of appropriate reporting guidelines for health-related research (see EQUATOR Network), life science research (see the BioSharing Information Resource), or the ARRIVE guidelines for reporting work involving animal research. Where applicable, authors should refer to any relevant reporting standards documents in this form.

If you have any questions, please consult our Journal Policies and/or contact us: editorial@elifesciences.org.

Sample-size estimation

- You should state whether an appropriate sample size was computed when the study was being designed
- You should state the statistical method of sample size computation and any required assumptions
- If no explicit power analysis was used, you should describe how you decided what sample (replicate) size (number) to use

Please outline where this information can be found within the submission (e.g., sections or figure legends), or explain why this information doesn't apply to your submission:

Replicates and sample size was estimated using extensive past experience of the techniques (e.g. PMIDs: 31995730, 31974009, 30100184, 29784826, 24094106 and 23911104) and no explicit power analysis was used. Sample sizes 'n' stated in the paper are from independent biological replicates (number of mice used, ranging from 3-16 depending on the experiments)

Replicates

- You should report how often each experiment was performed
- You should include a definition of biological versus technical replication
- The data obtained should be provided and sufficient information should be provided to indicate the number of independent biological and/or technical replicates
- If you encountered any outliers, you should describe how these were handled
- Criteria for exclusion/inclusion of data should be clearly stated
- High-throughput sequence data should be uploaded before submission, with a private link for reviewers provided (these are available from both GEO and ArrayExpress)

Please outline where this information can be found within the submission (e.g., sections or figure legends), or explain why this information doesn't apply to your submission:

Number of biological replicates used in the manuscript are stated in the individual figure legends. Mice used in behavior were excluded if the viral injection was not appropriately targeted. All data were included in the analysis, ruling out outliers. Electrophysiology data includes number of brain slices and number of cells used. The replicates are included in figure legends. Experiments for which data was averaged per mouse is stated below:
1) For Golgi staining experiments of LRRTM1/2-DKO mice used total of 65 dendrites for radiatum and 63 for outer molecular layer which were then averaged per mouse (Figure 2).
2) For Transmission electron microscopy experiments of LRRTM1/2-DKO mice, total of 144 micrographs for wildtype and 188 micrographs for DKO were used which were then averaged per mouse for number of synapses. Quantitative analysis of spine width used total of 759 synapses for WT and 590 synapses for DKO (Figure 2)
3) Confocal imaging for LRRTM1/2-cDKO and LRRTM1/2-DKO mice used total of 246-465 fields from 6 sections from 3-5 mice each (Figure 1,5 and figure supplement 3).

eLife Sciences Publications, Ltd is a limited liability non-profit non-stock corporation incorporated in the State of Delaware, USA, with company number 5030732, and is registered in the UK with company number FC030576 and branch number BR015634 at the address 1st Floor, 24 Hills Road, Cambridge CB2 1JP | August 2014

eLIFE
1st Floor
24 Hills Road
Cambridge CB2 1JP, UK
P 01223 855340
W elifesciences.org
T @elife

Statistical reporting

- Statistical analysis methods should be described and justified
- Raw data should be presented in figures whenever informative to do so (typically when N per group is less than 10)
- For each experiment, you should identify the statistical tests used, exact values of N, definitions of center, methods of multiple test correction, and dispersion and precision measures (e.g., mean, median, SD, SEM, confidence intervals; and, for the major substantive results, a measure of effect size (e.g., Pearson's r, Cohen's d)
- Report exact p-values wherever possible alongside the summary statistics and 95% confidence intervals. These should be reported for all key questions and not only when the p-value is less than 0.05.

Please outline where this information can be found within the submission (e.g., sections or figure legends), or explain why this information doesn't apply to your submission:

Information on statistical analysis has been included in the methods section as well as in detail in the figure legends for each individual graph. All the figures are represented as mean ± SEM. The graphs are given as aligned scatter plots with raw data along with bar graphs.

(For large datasets, or papers with a very large number of statistical tests, you may upload a single table file with tests, Ns, etc., with reference to sections in the manuscript.)

Group allocation

- Indicate how samples were allocated into experimental groups (in the case of clinical studies, please specify allocation to treatment method); if randomization was used, please also state if restricted randomization was applied
- Indicate if masking was used during group allocation, data collection and/or data analysis

Please outline where this information can be found within the submission (e.g., sections or figure legends), or explain why this information doesn't apply to your submission:

All experimental mice and samples were masked to experimenter during experiment and analysis.

Additional data files ("source data")

- We encourage you to upload relevant additional data files, such as numerical data that are represented as a graph in a figure, or as a summary table
- Where provided, these should be in the most useful format, and they can be uploaded as "Source data" files linked to a main figure or table
- Include model definition files including the full list of parameters used
- Include code used for data analysis (e.g., R, MatLab)
- Avoid stating that data files are "available upon request"

Please indicate the figures or tables for which source data files have been provided:

Raw data points are shown on all graphs. Imaging experiments show data as average from number of mice used. Number of sections, micrographs and neuronal dendrite processes used in analysis are stated in Replicates section of this form.
